# Functions of the Nervous System—The Cerebrum

**Published:** 1857-03

**Authors:** C. B. Chapman


### FUNCTIONS OF THE NERVOUS SYSTEM---THE CEREBRUM.

BY PROF. C. B. CHAPMAN, M. D.

In attempting to describe the nerve centres and their functions, as well as the nervous system as a whole, which I propose to do, in a short series of articles, it seems necessary in order that the account be at all complete, that we refer not only to the organs, but to some of their appendages.

There are three distinct nerve centres which perform diverse functions, besides those connected with the function of nutrition, which is the ganglionic or sympathetic system. These centres are the *cerebrum*, the *cerebellum*, the *spinal cord* and its appendage, the *medulla oblongata.* The spinal cord, or its type, exists in vertebrated animals, and is the centre of sensation and motion. Higher forms of animals of the same great class, have an organ, contained in a bony case at the upper or anterior extremity of the spinal cord, which secures an additional function, that of volition or ability for voluntary motion. This is the cerebrum, and may be regarded as the highest nerve centre, inasmuch as its function distinguishes the various grades of the higher animals, and its most exalted function distinguishes man from every other work of creation, by the possession of intelligence. The cerebellum performs a distinct function, although enclosed in the same bony case with the cerebrum. I can do no better in the attempt to carry out this plan than to describe the organs or instruments, and then refer to their various uses.

This article will be devoted to a description of the highest form of nerve centre, that which distinguishes man from the higher animals, in their highest grade of development.

The cerebrum, the cerebellum, and medulla oblongata, are enclosed in a group of eight bones, which are collectively called the cranium. The fourteen bones which constitute the face, are situated below the anterior portion of the cranium, and contain the principal organs of special sense. These two groups complete the collection of bones called the skull.

The cerebrum occupies seven-eighths of the cavity of the cranium, being seven times larger than the cerebellum. They are enclosed in three layers of membrane, which are 'quite diverse in character and function. The outer membrane is the dura mater. It is much thicker and more dense than the others; is of a fibrous character, and not only encloses the brain, but sends processes inward to divide and support different parts of the cerebral mass, and outward to form a sheath for the optic nerves. The processes which it sends inward are principally three in number, the *falx cerebri,* the *tentorium cerebelli* and *falx cerebelli.*

The falx cerebri is much the most extensive, as it separates the hemispheres into two equal parts by dipping down deep between them. It begins on the forward portion of the floor of the cranium, and extends forward by its upper border, then curves upward and backward, being in relation by its upper border with the middle of the frontal, then along the union of the two parietal bones at the sajgittal suture, then down the upper part of the occipital bone. It becomes broad toward its posterior termination, and rests upon the tentorium cerebelli, while along this attached border, a triangular opening is left between its layers, which constitutes the straight or fourth sinus.

The *tentorium* is a tent or roof of dura mater, thrown across the upper surface of the cerebellum, and attached to, or in relation with the posterior and lateral circumference of the posterior third of the inner surface of the cranium. The smallest appendage of the dura mater that passes inward, is the falx cerebelli. It is situated beneath the tentorium, and separates the cerebellar lobes.

The *pia mater* lies next to the convolutions of the brain, and is made up of small vessels, held together by fine cellular tissue. Its use seems to be to furnish the brain with a part of the blood demanded for its nutriment. Between the dura mater and the pia mater, is an attenuated double layer of membrane, which is enclosed at its borders, thus forming a shut sac. That enclosure is characteristic of serous membranes, to which class this belongs. At the attached border of the falx, and tentorium, and between the layers of these appendages of the dura mater, is transmitted the venous blood of the brain, which is received into the internal jugular vein as it escapes from the cavity of the cranium. These several vessels are called *sinuses.*

The cerebrum is divided by a deep fissure into two equal lateral halves. This fissure is occupied by the falx cerebri. The lateral halves are called hemispheres, each of which is subdivided into three unequal parts called anterior, middle and posterior lobes. The weight of the cerebrum is variable, ranging on subjects that are regarded as perfectly developed, from thirty-two to forty-eight ounces avoirdupois, the average being about two pounds and a half, the size or weight being somewhat varied by the general dimensions and weight of the individual.

I do not claim to have given more than an outline description of this important organ, but regard it as sufficient for our present purpose, which is, to describe its use in the economy of man.

The most that we know of its functions, like the general facts in physiology, is learned by comparison, that is, by comparing the functional activity of a cerebrum of one form and dimensions with another which differs from it. The absence of a cerebrum is found in all known examples to leave the being, however perfectly formed in its other organs, in the same condition with animals that possess only the spinal nerve centres; for the connecting organ which seems to combine the function of the cerebrum and spinal cord does not serve its usual purpose.

A very small cerebrum is found to be connected with an absence of capacity for the highest grade of intellectual culture, while a very large organ is not as uniformly connected with capacity for the highest culture; for it is found that in a few instances the idiot may possess a large, though an imperfectly developed cerebrum. We should keep in view that there is a well marked distinction between development and growth.

Human beings are sometimes born with an almost entire absence of cerebrum, and it is a fortunate incident that such beings are kindly spared a continued existence. Such children usually die young, commonly but a few hours after their birth. Exceptions to this rule are sometimes observed. One of the most interesting examples of which, on record, occurred at Hull, in England. In this instance, the being lived for several years, though it did not possess the ability to make known any of its natural wants—to make known even the return of necessity for nutriment. It was so low in the scale of animal existence, that it had no method of manifesting its desires, or of gratifying its wants, although the organs of mastication, and those of special sense, were not essentially defective. It was necessary, even when several years old, to prepare its food ready for stomach digestion, and then place it in relation with the organ of deglutition, when the reflex motion of the pharynx and œsophagus would convey the mass to the stomach.

We are able to refer to entirely opposite examples, showing at a glance the exalted function of the cerebrum, and to exhibit its capacity to perform, in ways that cannot admit of fallacy, when its usual stimulus to action is cut off. The most remarkable are those of Julia Brace, Oliver Carswell, and Laura Bridgman. Each of these persons was at an early age deprived of the use of the organs of sight and hearing; but when we study their characters and capacity separately, we will find an instructive lesson in the field of mental research. I will select the case of Laura Bridgman as the most interesting, from the circumstances in which she was early placed, as an example by which to illustrate the capacity and function of the cerebrum, when well developed, though deprived of the common avenues by which knowledge, even of the most simple kinds, is acquired.

There is, perhaps, no instance known to the civilized world that has excited more interest, as an example of psycological phenomena, and sympathy on account of her peculiar natural deprivations of the use of her most useful senses, than Laura Bridgman. Deprived as she is of sight and hearing, and consequently a mute, while she is reduced to the single avenue of the sense of touch as a medium of communication with the world of intelligence and matter, she has acquired the use of the manual alphabet, which she uses with nearly the same facility as those who can see, and is capable of receiving communications by this method with great rapidity.

I will here present a brief outline of the process of imparting instruction to her, and some facts with regard to the progress of her acquirements, which are compiled from Dr. Howe's reports of the Perkins Institution for the Blind, at South Boston, extending through some eighteen years.

Laura Bridgman was born at Hanover, New Hampshire, on the 21st of December, 1829. She is said to have been a very pretty infant, with bright blue eyes, but was puny and feeble until a year and a half old. She was then subject to spasms peculiar to that age of infancy, and probably occasioned by the eruption of the deciduous teeth, which seemed to rack her frame beyond the power of endurance; but after this period her health seemed better established, these dangerous paroxysms subsided, and at twenty months, she appeared perfectly well.

From this time her mental powers, which were before stinted, very rapidly developed themselves, and during four months of health which she enjoyed, making due allowance for a fond mother's account, she displayed a considerable degree of intelligence. But the hopes of her parents were soon doomed to disappointment; for she again suddenly sickened, and her disease raged with great violence during five weeks, when her eyes and ears inflamed and suppurated, and their contents were discharged. But the poor child's sufferings, although deprived of sight and hearing, were not ended. Her malady raged for seven weeks; and for five months she was kept in bed in a darkened room. It was a year before she could walk unsupported, and two years before she could sit up all day. When thus far recovered, it was observed that her sense of smell was almost entirely destroyed, and that of taste was much blunted. It was not until she was four years of age that her bodily infirmities did not preclude the possibility of her engaging in her apprenticeship of life and the world.

Let us reflect upon her situation at this period. The darkness and silence of the tomb were around her. No mother's smile or voice could awaken a response in the smile and prattle of the otherwise sprightly and vivacious child. No father's voice could reach her ear, and teach her to imitate its sound. All living nature was to her as though it were not, or were dead, save, perhaps, in the possession of motion and of varied temperature. So far was the poor creature degraded from the common condition of humanity, in possession of the usual avenues of sense and sensation, that the appellation, *thing*, seemed not inappropoiate in referring to her. Such, however, was not a faithful representation of her condition and person. The immortal spirit which had been implanted within her could not die, or even be maimed or mutilated. And though the usual avenues by which persons hold converse with the external world were cut off, this immortal nature began to manifest itself through others. She soon learned the form and outlines of the room she occupied, as well as the qualities of objects, such as form, density and temperature. She followed her mother in her occupations about the house, feeling her hands and arms, and then imitating her motions so accurately, as to learn to be useful in some employments, such as sewing and knitting. Her affections seemed early striving to expand and lavish themselves with peculiar force upon members of the family. Her means of communication and of perception were very limited. She was told to go to a given place by a gentle push with some specific sign, and to come to or to accompany one by a sign like drawing her; patting her gently on the head signified approbation, and on the back indicated disapprobation. She early manifested a strong disposition to learn, and began to express herself by a natural language of signs peculiarly original. She had a sign for each member of the family, which was suggested by some mark which her delicate sense of touch had revealed.

Laura first attracted the notice of Dr. Howe, who was then, and is at this time, Superintendent of the Perkins Asylum for the Blind, in South Boston, in 1837, being then nearly eight years of age ; and she was received into that institution in October of the same year. She was at first much bewildered with her new locality and associations ; and some two weeks were appropriated to giving her a knowledge of her new home. Then the task of instructing her in a knowledge of common objects and incidents was to commence. This was to be accomplished in one of two ways : either to go on and create, or build up, a new language of signs, on the basis of the natural language she had already commenced, to give her a sign for every individual thing ; or to give her a knowledge of letters, by a combination of which she could, like ourselves, receive and express her ideas and knowledge of a diversity of objects. It is at once apparent that the first would have been the easiest, but the least effectual. As she showed every disposition to learn, either method promised much more than would have been expected from a less attentive pupil. The first experiments were made by taking articles in common use, such as key, fork, spoon, etc., and putting labels upon them, with the name printed in raised letters. These she felt very carefully compared them, and soon learned that a different combination of characters stood for the different objects. Detached labels were then put into her hands, and she soon learned that they were similar to those attached to the articles. Then the individual letters were given her, on detached pieces of paper, from which she would arrange words, and place them upon the object or name they represented. During this year she attained great facility in the use of the manual alphabet.

The following is one of the plans adopted for the purpose of increasing her vocabulary: Her teacher gives her a new object, a pencil for instance, and allows her to examine it, and get an idea of its use; then she is taught how to spell its name by making the signs for the letters with her own fingers, while, as the different letters are formed, she turns her head a little to one side, like a person listening closely, her lips are apart, she seems scarcely to breathe, and her countenance, at first anxious, gradually changes to a smile as she comprehends the lesson. She then holds up her fingers, and spells the words in the manual alphabet, then she takes the types, and arranges the letters, and to make sure that she is right, takes the types composing the word, and places them in contact with the pencil. When left alone, she seems very happy, and will busy herself for hours in knitting and sewing. When without employment, she seems to amuse herself by imaginary dialogues, or by recalling past impressions; she spells out names of things she has recently learned, in the manual alphabet of the deaf mutes. In this lonely occupation she seems to reason, reflect and argue. If she spells a word wrong with the fingers of the right hand, she instantly strikes it with the left, as her teacher does, in token of disapprobation. When she is walking through a passage-way with her hands spread before her, she knows instantly those whom she meets, and passes them with a sign of recognition. If it be a girl of her own age, and especially one of her favorites, there is instantly a bright smile of recognition—there is a twining of arms, a grasping of hands; there are questions and answers, exchanges of expressions of joy and sorrow, there are kisses and caresses, just as between children who possess all their senses.

The first visit of Laura's mother to the Asylum was peculiarly interesting. The mother stood for some time, gazing with overflowing eyes upon her unfortunate child, who, all unconscious of her presence, was playing about the room.—Presently Laura ran against her, and at once began to feel her hands, examine her dress, and try to find out whether she knew her; but not succeeding in this, she turned away, as from a stranger, while the mother could no longer conceal the pang she felt at finding that her own beloved child did not know her. She next gave Laura a string of beads, which she used to wear at home, which were recognized by the child at once, who, with much joy, put them around her neck, and eagerly sought her teacher, to inform her that she understood the beads were from her home. The mother now tried to caress her child, but Laura repelled her, preferring to be with those with whom she was acquainted. Another article from her home was given her, and she began to look much interested. She examined the stranger more closely, and gave Dr. Howe to understand that she knew the person near her was from Hanover. She even endured her caresses, but would leave her with indifference, at the slightest signal. The distress of the mother was now painful to behold; for although she had feared that she should not be recognized, the painful reality of being treated with cold indifference by her darling child was too much for a mother's nature to bear.

After a while, on her mother taking hold of her again, a vague idea seemed to flit across Laura's mind that this could not be a stranger; she therefore eagerly felt her hands, while her countenance assumed an expression of intense interest; she became very pale, and then suddenly red, while hope seemed struggling with doubt and anxiety, and never were contending emotions more strongly depicted upon the human face.

At this moment of painful uncertainty, her mother drew close to her side, and kissed her fondly, when the truth flashed upon the child that it was her mother, and all mistrust and anxiety disappeared from her face, while, with an expression of exceeding joy, she eagerly nestled to the bosom of her parent, and yielded herself to her fond embraces. After this, the beads were all unheeded; the playthings which were offered her were utterly disregarded; her playmates, for whom but a moment before she gladly left the stranger, vainly strove to entice her from her mother, and though she yielded a ready obedience to the signal of her teacher to follow, it was evidently with painful reluctance. She clung close to her teacher as though bewildered and fearful, and when, after a brief suspense, she was taken to her mother, she clung to her with eager joy.

The subsequent parting of Laura's mother from her showed alike the affection, intelligence and resolution of the child. Laura accompanied her mother to the door, clinging close to her all the way until they arrived at the threshold, when she paused and felt around, in order to be certain who were near her. Perceiving the matron, of whom she was very fond, she grasped her with one hand, holding on convulsively to her mother with the other, and thus she stood for a moment; then she dropped her mother's hand, put her handkerchief to her eyes, turning round, clung sobbing to the matron, while the mother departed with emotions as deep as those of her child.

At this period, when but eleven years old, she seemed to possess a remarkable degree of conscientiousness, to duly respect the rights of others, and properly insist upon her own. It was early observed that she distinguished different degrees of intelligence in others, and that she regarded almost with contempt any new-comers, whenever she discovered in them marked weakness of mind. She chooses for her friends and companions those who are intelligent and can best talk with her, and evidently dislikes to be with those who are deficient in intellect, unless she can make them serve her purposes, which she is evidently inclined to do; for she takes advantage of such, making them wait upon her, thus indicating her Saxon blood.

She possesses a remarkable amount of fortitude, and seems ashamed to weep on account of pain, but not so when her sympathies are excited by the sufferings of her companions, or grief of her teacher. In such instances, her tears are frequent and unrestrained.

In her intellectual character, she manifests an insatiable thirst for knowledge, and a quick perception of the relation of things. She one day found a blank notice, printed in raised letters, directed to the trustees of the Asylum, in which the words "Sir," "trustees," "respectfully yrs," occurred. She ran eagerly to her teacher, asking the meaning of these words. She received the explanation of "sir and yours" without farther remark than that the latter was *like thine.* The explanation of "*respectfully*" did not seem easy to her teacher, and she was told that she must wait till after dinner. She seemed very sad, and said, "I will ask Doctor; I must know." When left alone one Sabbath, while the family were at Church, she was found quite excited when they returned, when it was discovered that she had found a book that contained a few Latin words in raised letters, which she had in vain tried to comprehend. She was only satisfied when told that her companions were mostly ignorant of the lauguage.

In her moral character, it is peculiarly gratifying to behold her continual gladness, her keen enjoyment of existence, her expansive love, her unhesitating confidence, her sympathy with suffering, as well as her conscientiousness, truthfulness and hopefulness. Her appreciation of language is accurate, according to her own mode, which is somewhat original; she is fond of detecting and of correcting errors, which sometimes affords her a rich fund for amusement. She is careful to distinguish number, and when some of her companions had the mumps, she perceived that they had a swelling on each side of the face. When she came to have the disease, and but one side being swollen, on being told that she had the mumps, she instantly corrected her informer by saying she had the mump.

There are few persons as active, and ready to imbibe every fact, as Laura. This activity is combined with a restlessness of temper that has required the most careful culture; for, sensitive as she is, any other than the mildest and most delicate rebuke could not be otherwise than injurious. Dr. Howe says he is confident that if, during the short explosions of temper that have manifested themselves, she had been treated with the slightest sternness, or even with coldness or indifference, the effect would have been most unfavorable. Her teacher, never for a moment losing her own temper, or ceasing to manifest the most tender interest in her pupil, has succeeded in having her judge and condemn herself, so that without being accused, she has perceived and corrected her fault, and without being punished, she has come out of the trial, stronger and better than before.

May we not infer that the teacher plays an humbler part in the work of intellectual development, than is commonly supposed? That his influence in forming the moral character, which gives direction to intellectual growth and pursuits, is great, can not be questioned. The story of Laura is full of instruction and pathos, affording a wide scope for thought, not only by the cultivator of science, but by the true philanthropist. A poor, blind, deaf and dumb girl, of humble hopes and humbler history, unconscious of being the center of so much regard, while almost every word and act is noted down and sought after by persons in various parts of the civilized world, with more interest than the story of the most conspicuous, noble born princess.

Let us reflect on what would be the impressions of Laura, if suddenly restored to her senses, like ourselves, and clothed with our faculties and intellect, which so far transcend her's. She would stand amazed to find herself the center of so much observation. She would look fearfully and anxiously back, to recall all her past thoughts and deeds, and perhaps painfully regret that some of them had not been better.

This account commences before Laura was eight years old. She is now twenty-six, and is still a boarder at the Asylum, where she enjoys social advantages which could not elsewhere be afforded her. She is of medium height, of very light complexion, slender; with well formed and delicately beautiful features. She is vivacious and cheerful as a lark, appearing to enjoy the pittance of life afforded her, more purely than most persons who possess all their senses. Every communication seems to convey a thrill of pleasure, that is manifested by a hearty laugh and quick response. Her muscular motions at such times seem to be but a vibration of the brain, in response to the most pleasing emotions.

There are few departments of science that present more difficulties in the way of accurate investigation, that are more complex than the study of the functions of the whole nervous system. It may seem a novel method to endeavor to illustrate an important group of facts in science, through the medium of a scientific journal, by a narrative as lengthy as the one here introduced. I have adopted this plan, in this instance, feeling confident that we have no other method by which we can convey so well, a proper idea of the functions of these important organs.

My next communication will be appropriated to an account of the functions of the spinal nerve center, and the influence which the cerebrum and the cerebellum are supposed to exercise over its actions, which constitutes collectively the cerebro-spinal function.

